# Comments on: fold change rank ordering statistics: a new method for detecting differentially expressed genes

**DOI:** 10.1186/s12859-016-1322-0

**Published:** 2016-11-15

**Authors:** Doulaye Dembélé, Philippe Kastner

**Affiliations:** 1 Institut de Génétique et de Biologie Moléculaire et Cellulaire (IGBMC), CNRS UMR 7104, INSERM U964, Université de Strasbourg, Illkirch, 67404 France; 2 IGBMC Microarray and Sequencing Platform, Illkirch, 67404 France; 3 Faculté de Medécine, Université de Strasbourg, Strasbourg, France

**Keywords:** Differentially expressed genes, Fold change, Average of ranks

## Abstract

We published a new method (BMC Bioinformatics 2014, 15:14) for searching for differentially expressed genes from two biological conditions datasets. The presentation of theorem 1 in this paper was incomplete. We received an anonymous comment about our publication that motivates the present work. Here, we present a complementary result which is necessary from the theoretical point of view to demonstrate our theorem. We also show that this result has no negative impact on our conclusions obtained with synthetic and experimental microarrays datasets.

## Background

To search for differentially expressed (DE) genes in profiling studies, we presented a new method based on fold change rank ordering statistics (FCROS). For the derivation of this method, we considered microarrays data from two biological conditions where *n* probes (genes) were used with *m*_1_ control and *m*_2_ test samples. We performed *k* pairwise comparisons (*k*=*m*_1_ *m*_2_) of the data samples and computed fold changes (FC) for each gene. The FCs obtained for each comparison were sorted in increasing order and their corresponding ranks were associated with genes. Hence, we can form a matrix of rank values *R* with components *r*_*ij*_ (*i*=1,2,…,*n*,*j*=1,2,…,*k*). We noted **r**_*i*_=[*r*_*i*1_ *r*_*i*2_ … *r*_*ik*_]^*T*^ the vector of rank values associated with gene *i*. We noted $\bar {r}_{i}$, the average of ranks (a.o.r) value for gene *i*. The value for $\bar {r}_{i}$ varies between $a=\min _{i}\{\bar {r}_{i}\}$ and $b=\max _{i}\{\bar {r}_{i}\}$. That allows to associate an unique vector of a.o.r values with the *n* genes: $\bar {\mathbf {r}}=[a,\ (a+\delta _{1}),\ (a+\delta _{1}+\delta _{2}),\ \ldots,\ (a+\delta _{1}+\ldots +\delta _{n-2}),\ b]^{T}$ where the scalars *δ*_*i*_ are the differences between consecutive ordered a.o.r. Without loss of generality, we assumed that the differences *δ*_*i*_ have the same value which is approximated by their mean: $\delta =\frac {b-a}{n-1}$. Using these notations, we derived a theorem showing a normal distribution for vector $\bar {\mathbf {r}}$ [1]. The content of this theorem was incomplete as shown in the following lemma we received from an anonymous reader.

### **Lemma 1**

Let consider the matrix of rank values *R* under the assumption that the rank values in each column are all distinct. Assume uniform random sampling without replacement model for the columns of *R*, i.e. each column of *R* is an independent draw from the set of all permutations of {1,…,*n*} with uniform probability $\frac {1}{n!}$ for each permutation. Then, the asymptotic distribution of the unordered vector average of rank (a.o.r.), $r=\left (r_{i}=\frac {1}{k}\sum _{j=1}^{k}R_{ij}\right), i\in 1\ldots n$, has a mean $\frac {n+1}{2}\mathbf {1}_{n}$ and degenerate variance-covariance matrix *Σ*(*n*,*n*), det*Σ*=0:

1 $$\begin{array}{@{}rcl@{}} \Sigma = \left(\begin{array}{ccccc} \beta & \alpha & \ldots & \alpha & \alpha\\ \alpha & \beta & \ldots & \alpha & \alpha\\ \vdots & \vdots & \ddots & \vdots & \vdots\\ \alpha & \alpha & \ldots & \beta & \alpha\\ \alpha & \alpha & \ldots & \alpha & \beta \end{array}\right)  \end{array} $$

with diagonal element $\beta =\frac {n^{2}-1}{12}$, off-diagonal element $\alpha =-\frac {\beta }{n-1}$ and **1**_*n*_=[1,1,…,1]^*T*^.

### *Proof*

Note that for *k*→*∞*, the appearance of all elements of the set {1,…,*n*} in each row of *R* under the assumed sampling model are equally likely, hence by the weak law of large numbers ([2], page 235) the asymptotic mean is the constant vector $\left (\frac {1}{n}\sum _{i=1}^{n}i\right)\mathbf {1}_{n} =\frac {n+1}{2}\mathbf {1}_{n}$. Under the same observation, the asymptotic variance, ∀*ℓ*∈{1,…,*n*}, is equal to:

2 $$\begin{array}{@{}rcl@{}}  Var\left(r_{\ell}\right) \underset{k}{\longrightarrow} \beta & = &\frac{1}{n}\left[\sum_{i=1}^{n} \left(i-\frac{n+1}{2}\right)^{2}\right] = \frac{n^{2}-1}{12}  \end{array} $$

The asymptotic covariance is computed as a two-index summation over the set {1,…,*n*} with the restriction that no two indices can be the same since the columns are permutations by construction, hence ∀*ℓ*≠*m*∈{1,…,*n*}:

3 $$\begin{array}{@{}rcl@{}} &&Cov\left(r_{\ell},r_{m}\right)\underset{k}{\longrightarrow}\alpha\\ &&= \!\frac{1}{n\left(n-1\right)}\sum_{i=1}^{n}\sum_{\substack{j=1\\ j\neq i}}^{n}\left(i-\frac{n+1}{2}\right)\left(j-\frac{n+1}{2}\right) \end{array} $$

4 $$\begin{array}{@{}rcl@{}} && = \! \frac{1}{n\left(n-1\right)}\!\left\{\!\!\left[\!\sum_{i=1}^{n}\! \left(\!i\,-\,\frac{n+1}{2}\!\right)\!\!\right]^{2}\!\,-\,\sum_{i=1}^{n}\!\! \left(\!i\,-\,\frac{n+1}{2}\right)^{2}\right\} \end{array} $$

5 $$\begin{array}{@{}rcl@{}} && = \! -\frac{1}{n\left(n-1\right)}\sum_{i=1}^{n} \left(i-\frac{n+1}{2}\right)^{2}=-\frac{\beta}{n-1}. \end{array} $$

Thus, since *Σ* **1**_*n*_=**0**, it follows that det*Σ*=0. □

This lemma shows that the covariance term was missed in our theorem. In the next section, we present a complete version of our theorem using the notations we adopted in [1].

## Results

From our notations, we have $\bar {\mathbf {r}} = [a,\ a+\delta,\ a+2\delta,\ \ldots,\ a+(n-1)\delta ]^{T}$ the vector with the a.o.r values. Each component of the vector $\bar {\mathbf {r}}$ can be writen as: $\mathcal {R}_{\ell }=(a+\ell \delta), \ell =0, 1, \ldots, n-1$. The theorem 1 in ([1], page 3) should be read as:

### **Theorem 1**

When the number *k* of the pairwise comparisons grows, the ordered average of ranks (a.o.r.) $\bar {\mathbf {r}}$ have a normal distribution. The mean of this distribution is $\frac {a+b}{2}\mathbf {1}_{n}$, its variance-covariance matrix has diagonal element $\frac {n^{2}-1}{12}\delta ^{2}$ and off-diagonal element $-\frac {n+1}{12}\delta ^{2}$, where *a* and *b* are the minimum and the maximum of the observed a.o.r., $\bar {\mathbf {r}}$, respectively. *δ* is the average difference between consecutive ordered a.o.r. $\bar {\mathbf {r}}$.

### *Proof*

From the following definitions:

$$\begin{array}{@{}rcl@{}} E\{\mathcal{R}_{\ell}\} &=& \frac{1}{n}\sum_{\ell=1}^{n}\mathcal{R}_{\ell}\\ Var(\mathcal{R}_{\ell}) &=& E\{\mathcal{\mathcal{R}}_{\ell}^{2}\} - \left(E\{\mathcal{R}_{\ell}\}\right)^{2}\\ Cov(\mathcal{R}_{\ell},\mathcal{R}_{m})_{m\neq \ell} &=& E\{\mathcal{R}_{\ell} \mathcal{R}_{m}\} - \left(E\{\mathcal{R}_{\ell}\}\right)^{2} \end{array} $$

and using $\delta = \frac {b-a}{n-1}$, a component of the mean of the normal distribution is:

6 $$  E\left\{\sum_{\ell=0}^{n-1}(a+\ell\delta)\right\} = \frac{1}{n}\sum_{\ell=0}^{n-1} (a+\ell\delta) = a+\frac{n-1}{2}\delta = \frac{b+a}{2}.   $$

A component of the variance (diagonal element) of the normal distribution matrix is:

7 $$ {\begin{aligned} Var(\mathcal{R}_{\ell}) &= E\left\{\sum_{\ell=0}^{n-1}(a+\ell\delta)^{2}\right\} - \left(a+\frac{n-1}{2}\delta\right)^{2}\\ &= E\left\{\sum_{\ell=0}^{n-1}\left(a^{2}+2a\delta\ell+\delta^{2}\ell^{2}\right)\right\} - \left(a+\frac{n-1}{2}\delta\right)^{2}\\ &= \frac{1}{n}\left(na^{2}+2a\delta\frac{n(n-1)}{2} + \frac{n(n-1)(2n-1)}{6}\delta^{2}\right)\\ &\quad- \left(a+\frac{n-1}{2}\delta\right)^{2} = \frac{n^{2}-1}{12}\delta^{2}. \end{aligned}}  $$

A component of the covariance (off-diagonal element) of the normal distribution matrix is:

8 $$ \begin{aligned} Cov(\mathcal{R}_{\ell},\mathcal{\mathcal{R}}_{m})_{m\neq \ell} &= E\left\{\sum_{\ell=0}^{n-1}\sum_{\substack{m=0\\ m\neq \ell}}^{n-1} (a+\ell\delta)(a+m\delta)\right\} - \left(a+\frac{n-1}{2}\delta\right)^{2}\\ &= E\left\{\sum_{\ell=0}^{n-1}\sum_{m=0}^{n-1} \left(a^{2}+a\delta\ell+a\delta m +\delta^{2}m\ell\right)\right.\\& \left.\qquad-\sum_{\ell=0}^{n-1}(a+\ell\delta)^{2}\right\} - \left(a+\frac{n-1}{2}\delta\right)^{2}\\ &= \frac{1}{n(n-1)}\left(n^{2}a^{2}+n^{2}(n-1)a\delta+\frac{n^{2}(n-1)^{2}}{4} \delta^{2}\right.\\ & \left. -na^{2}-n(n-1)a\delta-\frac{n(n-1)(2n-1)}{6}\delta^{2}\right)\\ &\quad- \left(a+\frac{n-1}{2}\delta\right)^{2}= -\frac{n+1}{12}\delta^{2}. \end{aligned}  $$

□

By setting *a*=*δ*=1 and *b*=*n* in the theorem 1, the mean and the variance-covariance component values are the same as in lemma 1. These setting values for *a*,*b* and *δ* correspond to the case we called ideal situation ([1], page 4).

For the FCROS algorithm, we used the standardized rank value, i.e., each observed rank value is divided by *n*. The mean and variance-covariance components should be divided by *n* and *n* ^2^ respectively. This leads to a mean component $r^{\star }=\left (\frac {1}{2}+\frac {1}{2n}\right)$, and a variance-covariance matrix with a diagonal component $\beta ^{\star }=\left (\frac {1}{12}-\frac {1}{12n^{2}}\right)$ and a off-diagonal component $\alpha ^{\star }=-\left (\frac {1}{12}-\frac {1}{12n^{2}}\right)\frac {1}{n-1}$. Table 1 shows the values for *r* ^⋆^,*β* ^⋆^ and *α* ^⋆^ when *n* increases. For a large value for *n*, the off-diagonal components of the variance-covariance matrix vanish. Hence, when *n* is large, a good approximation for the mean and the variance components are $\frac {1}{2}$ and $\frac {1}{12}$, respectively.

**Table 1:** Values of the mean, the variance and the covariance components when *n* increases

n	10	100	1,000	10,000
*r* ^⋆^	$\frac {1}{2}+5*10^{-2}$	$\frac {1}{2}+5*10^{-3}$	$\frac {1}{2}+5*10^{-4}$	$\frac {1}{2}+5*10^{-5}$
*β* ^⋆^	$\frac {1}{12}-8.33*10^{-4}$	$\frac {1}{12}-8.33*10^{-6}$	$\frac {1}{12}-8.33*10^{-8}$	$\frac {1}{12}-8.33*10^{-10}$
*α* ^⋆^	−9.17∗10^−3^	−8.4∗10^−4^	−8.34∗10^−5^	−8.33∗10^−6^

## Discussion and conclusions

As shown, the theorem we previously presented was incomplete since the covariance term was missed. The present complementary result is necessary from the theoretical point of view, and we are grateful to the anonymous reader for pointing this out. This result will be useful for small values of *n*. However, for high throughput biological datasets, *n* is large, often greater than 10,000 ([1], page 2). For such values of *n*, the rank deficient variance-covariance matrix of the normal distribution associated with the a.o.r values is near a diagonal matrix. Hence, it is as if the a.o.r values of each gene follow a normal distribution with parameters $\frac {1}{2}$ and $\frac {1}{12}$.
